# Association Between Liver Enzymes and Metabolic Syndrome: A Population‐Based Cross‐Sectional Study of the Bandar Kong Cohort Study

**DOI:** 10.1155/ijh/8920064

**Published:** 2026-04-08

**Authors:** Arezoo Ghazalgoo, Masoumeh Kheirandish, Seyyed Mohammad Hashemi, Elaheh Salarpour, Ayoub Basham, Parsa Saberian, Ehsan Amini-Salehi, Hoda Safa

**Affiliations:** ^1^ Department of Endocrinology, Endocrinology and Metabolism Research Center, Hormozgan University of Medical Sciences, Bandar Abbas, Iran, hums.ac.ir; ^2^ Department of Cardiology, Cardiovascular Research Center, Hormozgan University of Medical Sciences, Bandar Abbas, Iran, hums.ac.ir; ^3^ Student Research Committee, Faculty of Medicine, Hormozgan University of Medical Sciences, Bandar Abbas, Iran, hums.ac.ir; ^4^ Gastrointestinal and Liver Diseases Research Center, Guilan University of Medical Sciences, Rasht, Iran, gums.ac.ir; ^5^ Department of Gastroenterology and Hepatology, Hormozgan University of Medical Sciences, Bandar Abbas, Iran, hums.ac.ir

**Keywords:** alanine transaminase, alkaline phosphatase, aspartate transaminase, cross-sectional study, gamma-glutamyl transferase, metabolic syndrome

## Abstract

**Background:**

Metabolic syndrome (MetS) is a major global health concern that increases morbidity and mortality from cardiometabolic diseases. We refined the rationale to highlight the mechanistic involvement of the liver in metabolic dysregulation.

**Methods:**

This population‐based study was conducted on 3896 adults aged between 35 and 70 (57.2% female) who participated in the Bandare‐Kong Non‐Communicable Diseases (BKNCD) cohort study. Participants were categorized into normal quartiles and abnormal levels of liver enzymes including gamma‐glutamyl transferase (GGT), alanine transaminase (ALT), aspartate transaminase (AST), and alkaline phosphatase (ALP). MetS was defined based on the modified National Cholesterol Education Program Adult Treatment Panel III (NCEP ATP III) criteria for Iranian population. Logistic regression was used to calculate odds ratios (ORs) with 95% confidence intervals (CIs) and *p* values, adjusting for age, sex, residence, BMI, physical activity, energy intake, and financial status.

**Results:**

The prevalence of MetS was 36.6%. After multivariable adjustment, abnormal levels of GGT (OR = 1.91, 95% CI: 1.61–2.28, *p* < 0.001), ALT (OR = 1.60, 95% CI: 1.34–1.90, *p* < 0.001), ALP (OR = 1.48, 95% CI: 1.07–2.03, *p* = 0.01), and AST (OR = 1.39, 95% CI: 1.09–1.77, *p* = 0.007) were significantly associated with higher odds of MetS. Within normal ranges, the highest quartile of GGT (OR = 2.81, 95% CI: 2.22–3.56, *p* < 0.001), ALT (OR = 2.39, 95% CI: 1.89–3.03, *p* < 0.001), and ALP (OR = 1.63, 95% CI: 1.30–2.04, *p* < 0.001) showed significant associations, while AST did not.

**Conclusion:**

Serum liver enzyme levels, including those within normal range, were strongly associated with MetS. GGT showed relatively stronger associations compared with other enzymes; however, these findings should not be interpreted as diagnostic superiority because no performance metrics (AUC, PPV, and NPV) were evaluated. ALT and ALP also showed meaningful associations. Future studies should assess diagnostic accuracy before considering clinical implementation.

## 1. Introduction

Metabolic syndrome (MetS) is defined as a cluster of metabolic abnormalities—central obesity, dyslipidemia, glucose intolerance, and elevated blood pressure—that together lead to markedly increased cardiovascular and metabolic risk [1, 2]. Its prevalence varies globally from 14% to 32% [3] and in Iran reaches nearly 30%, with even higher burdens in southern coastal regions [4, 5]. Elevated liver enzymes are well‐established correlates of metabolic dysfunction and cardiovascular risk [6, 7]. In particular, prospective studies have identified higher alanine aminotransferase (ALT) and gamma‐glutamyl transferase (GGT) levels as significant predictors of future cardiovascular events [8].

Several studies found bidirectional association between MetS and elevated liver enzymes [9–12].

A meta‐analysis of 17 studies involving 76,686 individuals with MetS and 201,855 controls found that participants with MetS had significantly higher plasma levels of ALT, aspartate aminotransferase (AST), and GGT compared with their counterparts [13]. Furthermore, other investigations have shown that elevated ALT and AST concentrations increase the likelihood of developing MetS [14–16]. Notably, evidence suggests that even “normal‐range” liver enzyme values may carry metabolic significance, yet this remains incompletely understood [17].

Although there is a general agreement that abnormal liver enzyme levels signal an increased risk of MetS [13–16], the predictive utility of normal‐range values remains uncertain, and comparative performance across enzymes, particularly the strong signal repeatedly observed for *γ*‐glutamyl transferase, has not been adequately evaluated. Therefore, this study was designed to examine the association of MetS with liver enzyme levels across both normal and abnormal ranges.

## 2. Method

### 2.1. Design and Participants

This cross‐sectional study utilized baseline data from the Bandare‐Kong Non‐Communicable Diseases (BKNCD) cohort, a component of the Prospective Epidemiological Research Studies in IrAN (PERSIAN) project, conducted under the supervision of the Iranian Ministry of Health and Medical Education. A total of 4040 residents of Bandar‐e Kong City, aged 35–70 years, were recruited. We excluded participants with incomplete data, alcohol consumption, pregnancy, or any chronic diseases including heart disease, viral hepatitis, chronic liver disease, chronic kidney disease, thyroid disorders, hemochromatosis, Wilson′s disease, autoimmune diseases, and cancer. Individuals taking medications or supplements that affect liver enzymes (such as contraceptives, glucocorticoids, progestins, spironolactone, thiazolidinediones, ursodeoxycholic acid, interferon, tamoxifen, amiodarone, or antituberculosis drugs) were also excluded. A total of 3896 participants remained after applying exclusion criteria.

### 2.2. Data Collection and Measurements

A total of 4040 residents of Bandar‐e Kong City voluntarily enrolled in the baseline phase of the cohort study. All the procedures, including interview, physical examination, and collecting biosamples, were performed under the supervision of the PERSIAN Cohort inspection team. The validity and reliability of the questionnaires used in the cohort have been previously published [18, 19]. Questionnaire‐based data, including demographic, medical records, daily energy intake, and physical activity, were collected during a face‐to‐face interview. Weight was assessed using a standard clinical scale while participants wore light clothing and were barefoot. Height was measured with a standard stadiometer, also while barefoot. Body mass index (BMI) was calculated by dividing weight (in kilograms) by height squared (in meters squared). Waist circumference (WC) was measured at the midpoint between the iliac crest and the lowest rib using a flexible tape measure; it was calculated twice per subject, and the average was recorded. Blood pressure was measured by a sphygmomanometer according to standards. Blood pressure was measured twice, 15 min apart, following a 5‐min seated rest, and the mean was recorded as systolic blood pressure (SBP) and diastolic blood pressure (DBP). Venous blood samples were collected after a 12‐h overnight fast, stored at −80°C, and analyzed for complete blood count (CBC), fasting blood glucose (FBS), lipid profile, and liver enzymes using standardized enzymatic kits (Pars Azmoun Co., Tehran, Iran).

### 2.3. Variables and Definitions

Participants were classified as normal or abnormal for each liver enzyme according to the reference ranges provided by the standard kit (Table S1).

To assess the relationship between liver enzyme levels within the normal range and MetS, liver enzymes (ALT, AST, ALP, and GGT) were categorized as follows:

1. Normal versus abnormal according to kit‐defined reference ranges
2. Normal‐range quartiles (Q1–Q4)

MetS was defined based on the modified NCEP ATP III criteria for Iranian adults; individuals who meet at least three of the five criteria are diagnosed with MetS [20]:

1. Central obesity: WC of 95 cm and above is considered central obesity in males and females in the Iranian population [21].
2. Hypertriglyceridemia: a fasting blood triglyceride level of 150 mg/dL and above or receiving medication for high blood triglyceride levels.
3. Low plasma levels of high‐density lipoprotein cholesterol (HDL‐c): HDL‐c of less than 40 mg/dL in males or less than 50 mg/dL in females.
4. Hypertension: SBP of 130 mmHg and above or DBP of 85 mmHg and above or taking medication for hypertension.
5. Hyperglycemia: FBS of 100 mg/dL and above or those who are taking medication for hyperglycemia or are diabetic.

Physical activity was evaluated using the International Physical Activity Questionnaire (IPAQ) and expressed as metabolic equivalent of task (MET)‐minutes per week. Participants were categorized as having low (24–36.5 METs), moderate (36.6–44.9 METs), or high (≥ 45 METs) physical activity levels. Daily energy intake was assessed using a validated semiquantitative food frequency questionnaire (FFQ) specifically adapted for the study population. Energy calculations were performed using Nutritionist IV software (First Databank Inc., United States) updated with Iranian food composition tables. Energy intake was expressed in kilocalories per day.

### 2.4. Data Analysis

Data analysis was performed using SPSS Version 25. Continuous variables are presented as mean ± standard deviation (SD) and categorical variables as frequency and percentage. Normality of data distribution was assessed using the Kolmogorov–Smirnov test. Independent *t*‐tests and chi‐square tests were used to compare continuous and categorical variables between groups, respectively. Binary logistic regression was applied to estimate odds ratios (ORs) for abnormal liver enzyme levels in relation to MetS. To assess associations within the normal range of liver enzymes, participants were categorized into quartiles (Q1–Q4), with Q1 as the reference, and multinomial logistic regression was used to calculate ORs for each quartile. Crude analyses are presented as Model 1. Model 2 was adjusted for potential confounders selected based on a combination of statistical criteria (*p* < 0.20 in crude analysis) and biological plausibility, including age, sex, BMI, physical activity, and smoking. Directed acyclic graphs (DAGs) were used when applicable to confirm the minimal sufficient set of confounders. Multicollinearity among liver enzymes was assessed using variance inflation factors (VIFs). All VIFs were below 5, indicating that multicollinearity was not a concern in the multivariable models. Sensitivity analyses using separate models for each enzyme confirmed the robustness of the results. All tests were two‐sided, and a *p* value < 0.05 was considered statistically significant. Because the dataset did not include sex‐stratified distributions of liver enzyme levels, we were unable to compute sex‐specific descriptive values (means or prevalence of abnormality). Therefore, sex‐specific comparisons were not analyzed inferentially, and any discussion of sex differences was restricted to qualitative interpretation based on existing literature.

## 3. Result

### 3.1. Descriptive Data

Of the 4040 participants, 3896 met the inclusion criteria (1671 men [42.8%] and 2225 women [57.2%]). Most participants (85.1%) resided in urban areas, and 39.8% were in the top two quintiles of annual income. Approximately 25.6% had insufficient physical activity. The prevalence of MetS in the study population was 36.6% (*n* = 1426) (Tables 1 and 2). Although sex differences in metabolic profiles are well documented, sex‐stratified liver enzyme data were not available in the current dataset; therefore, descriptive enzyme patterns could not be examined separately for men and women.

**Table 1:** Baseline characteristics of participants according to metabolic syndrome status.

Variable	MetS yes (*n* = 1426)	MetS no (*n* = 2470)	*p* value
Age (years)	51.54 ± 9.29	46.45 ± 8.92	< 0.001
35–44 (%)	24.0	76.0	< 0.001
45–54 (%)	37.7	62.3	
55–70 (%)	53.9	46.1	
Male (%)	31.4	68.6	< 0.001
Female (%)	40.5	59.5	
Urban (%)	35.9	64.1	0.02
Rural (%)	40.8	59.2	
Physical activity low (%)	42.7	57.3	< 0.001
Moderate (%)	36.6	63.4	
High (%)	25.1	74.9	
BMI (kg/m^2^)	29.31 ± 4.57	25.54 ± 4.71	< 0.001
Energy intake (kcal/day)	2851.84 ± 930.78	2914.41 ± 928.37	0.04

**Table 2:** Liver enzyme profiles by metabolic syndrome status.

Liver enzyme variable	MetS yes (%)	MetS no (%)	*p* value
ALT abnormal	31.7	23.8	< 0.001
ALT Q1	22.3	77.7	< 0.001
ALT Q2	33.7	66.3	
ALT Q3	37.6	62.4	
ALT Q4	43.0	57.0	
AST abnormal	42.7	57.3	0.007
AST Q1	37.7	62.3	0.001
AST Q2	33.7	66.3	
AST Q3	33.0	67.0	
AST Q4	40.4	59.6	
ALP abnormal	51.2	48.8	< 0.001
ALP Q1	25.7	74.3	< 0.001
ALP Q2	34.1	65.9	
ALP Q3	37.5	62.5	
ALP Q4	46.2	53.8	
GGT abnormal	49.9	50.1	< 0.001
GGT Q1	17.8	82.2	< 0.001
GGT Q2	28.2	71.8	
GGT Q3	37.3	62.7	
GGT Q4	48.1	51.9	

*Note:* Values are presented as mean ± SD or *n* (%). Quartiles (Q1–Q4) were defined among participants with enzyme levels within sex‐specific normal ranges. *p* values were calculated using independent *t*‐test or chi‐square test.

Abbreviations: ALP, alkaline phosphatase; ALT, alanine aminotransferase; AST, aspartate aminotransferase; BMI, body mass index; GGT, gamma‐glutamyl transferase; MET, metabolic equivalent of task; MetS, metabolic syndrome.

### 3.2. MetS and Disturbance in Liver Enzymes

After adjustment for confounding variables including age, sex, place of residence, physical activity, socioeconomic status, BMI, energy intake, and liver enzyme levels, the results showed that abnormal ALT was associated with a 60% higher risk of MetS (OR = 1.60; 95% CI: 1.34–1.90; *p* < 0.001). Likewise, abnormal levels of other enzymes were also associated with a higher likelihood of MetS, with a 39% increase for AST (OR = 1.39; 95% CI: 1.09–1.77; *p* = 0.007), a 48% increase for ALP (OR = 1.48; 95% CI: 1.07–2.03; *p* = 0.01), and nearly a twofold increase for GGT (OR = 1.91; 95% CI: 1.61–2.28; *p* < 0.001), respectively (Table 3).

**Table 3:** The association between abnormal liver enzymes and metabolic syndrome.

Variable	Model 1		Model 2	
	OR (95% CI)	*p* value	OR (95% CI)	*p* value
ALT	1.57 (1.36–1.82)	< 0.001	1.60 (1.34–1.90)	< 0.001
AST	1.33 (1.08–1.64)	0.007	1.39 (1.09–1.77)	0.007
ALP	1.88 (1.42–2.49)	< 0.001	1.48 (1.07–2.03)	0.01
GGT	2.07 (1.78–2.41)	< 0.001	1.91 (1.61–2.28)	< 0.001

*Note:* Reference: normal liver enzyme. Model 1 represents a crude analysis, while Model 2 incorporates adjustments for variables including age, gender, place of residence, physical activity, financial status, body mass index, energy intake, and liver enzyme levels.

Abbreviations: ALP, alkaline phosphatase; ALT, alanine transaminase; AST, aspartate transaminase; GGT, gamma glutamyl transferase.

### 3.3. MetS and Liver Enzymes Within the Normal Range

In multivariable analysis (Model 2), participants in the second, third, and fourth quartiles of ALT within the normal range had 1.52 (95% CI: 1.16–2.00, *p* = 0.002), 1.85 (95% CI: 1.42–2.42, *p* < 0.001), and 2.39 (95% CI: 1.89–3.03, *p* < 0.001) times higher odds of MetS, respectively, compared with the first quartile. A similar dose–response trend was observed for ALP, with ORs of 1.27 (95% CI: 1.01–1.59, *p* = 0.01), 1.33 (95% CI: 1.06–1.66, *p* < 0.001), and 1.63 (95% CI: 1.30–2.04, *p* < 0.001) for Q2, Q3, and Q4, respectively. GGT showed the strongest association, with ORs of 1.35 (95% CI: 1.02–1.79, *p* = 0.03), 1.84 (95% CI: 1.41–2.41, *p* < 0.001), and 2.81 (95% CI: 2.22–3.56, *p* < 0.001) for Q2, Q3, and Q4, respectively. In contrast, AST in the normal range was not significantly associated with MetS (OR for Q4 vs. Q1 = 1.11, 95% CI: 0.89–1.37, *p* = 0.35), indicating it may be less informative as a marker within normal limits. Overall, while GGT had the highest point estimate, ALT and ALP also showed substantial associations, suggesting that multiple liver enzymes are relevant for MetS risk even within the normal range.

While GGT consistently demonstrated comparatively larger effect‐size estimates, these differences should not be interpreted as evidence of diagnostic superiority (Table 4).

**Table 4:** The association between liver enzymes within the normal range and metabolic syndrome.

Variable	Model 1		Model 2	
	OR (95% CI)	*p* value	OR (95% CI)	*p* value
ALT				
Q1	1	—	1	—
Q2	1.77 (1.39–2.24)	< 0.001	1.52 (1.16–2.00)	0.002
Q3	2.10 (1.67–2.65)	< 0.001	1.85 (1.42–2.42)	< 0.001
Q4	2.63 (2.15–3.21)	< 0.001	2.39 (1.89–3.03)	< 0.001
AST				
Q1	1	—	1	—
Q2	0.84 (0.69–1.02)	0.08	0.88 (0.70–1.11)	0.28
Q3	0.81 (0.67–0.99)	0.04	0.80 (0.64–1.01)	0.06
Q4	1.12 (0.94–1.34)	0.21	1.11 (0.89–1.37)	0.35
ALP				
Q1	1	—	1	—
Q2	1.50 (1.23–1.83)	< 0.001	1.27 (1.01–1.59)	0.01
Q3	1.74 (1.43–2.12)	< 0.001	1.33 (1.06–1.66)	< 0.001
Q4	2.49 (2.05–3.02)	< 0.001	1.63 (1.30–2.04)	< 0.001
GGT				
Q1	1	—	1	—
Q2	1.82 (1.42–2.33)	< 0.001	1.35 (1.02–1.79)	0.03
Q3	2.75 (2.16–3.49)	< 0.001	1.84 (1.41–2.41)	< 0.001
Q4	4.28 (3.47–5.28)	< 0.001	2.81 (2.22–3.56)	< 0.001

*Note:* Model 1 represents a crude analysis, while Model 2 incorporates adjustments for variables including age, gender, place of residence, physical activity, financial status, body mass index, energy intake, and liver enzyme levels.

Abbreviations: ALP, alkaline phosphatase; ALT, alanine transaminase; AST, aspartate transaminase; GGT, gamma glutamyl transferase.

## 4. Discussion

The main objective of this study was to evaluate the relationship between liver enzyme concentrations both above and within the normal range and MetS in a large Middle Eastern population. We found that elevated levels of GGT, ALT, ALP, and AST were significantly associated with higher odds of MetS, with GGT demonstrating the strongest association. Importantly, even among individuals with enzyme concentrations within reference limits, those in the highest quartiles of GGT, ALT, and ALP showed substantially greater likelihood of MetS, indicating that early metabolic disturbances may be detectable before clinical thresholds are crossed. A large case–control investigation from China (*n* ≈ 12,500) reported that higher concentrations of GGT, ALT, AST, and ALP were strongly associated with increased odds of MetS, with GGT showing the greatest effect size [22]. Similarly, studies from Korea and Japan have demonstrated that elevated ALT and GGT levels are independently linked with a higher prevalence of MetS [14, 23, 24]. Data from the Rafsanjan cohort in Iran further support this relationship, showing progressively higher liver enzyme levels among individuals with multiple metabolic abnormalities, indicating a dose–response pattern [16]. Together, these findings suggest that liver enzymes reflect underlying metabolic derangements and may also correlate with disease severity. Despite these consistent trends, some heterogeneity exists among populations. For example, the PERSIAN Guilan cohort reported that AST lost statistical significance after full adjustment, whereas ALT and GGT remained independent predictors of MetS [25]. Such discrepancies may arise from regional differences in demographics, lifestyle, genetic predisposition, laboratory thresholds, or residual confounding, emphasizing the need for population‐specific interpretation of liver enzyme values.

Importantly, the association between liver enzymes and MetS extends well into the normal laboratory range. Several investigations including those by Zhang et al. and Korean population studies have shown that higher normal levels of GGT and ALT significantly predict MetS [22]. A Korean population‐based study also demonstrated that ALT and AST retained predictive capacity for MetS even within standard laboratory ranges [14]. A study from East Azerbaijan, Iran, highlighted potential sex‐based patterns, reporting notably higher normal ALT and AST levels among women with multiple metabolic abnormalities; the highest ALT quartile conferred a fourfold higher risk of MetS in women [26]. Conversely, some studies, including that by Chen et al., found no association between normal‐range AST and MetS [27], a finding mirrored in our own analysis. Such inconsistencies likely reflect differences in age distribution, hormonal influences, genetic background, alcohol exposure, or medication use.

Consistent with our findings, a matched case–control study in northern Iran reported that ALT levels—whether mildly elevated or high‐normal—were consistently associated with increased MetS risk, while AST showed no predictive utility [15].

These observations collectively imply that current laboratory reference intervals for liver enzymes may overlook individuals at an early metabolic risk. This has prompted several expert groups to recommend lowering sex‐specific upper normal limits for key liver enzymes [28].

The biological plausibility of our results is supported by well‐established mechanisms. ALT, a cytosolic hepatocellular enzyme, rises even in mild steatosis and is a recognized surrogate for NAFLD and hepatic insulin resistance [29]. NAFLD, in turn, has well‐documented associations with obesity, hypertension, dyslipidemia, and impaired glucose metabolism [30–32]. These overlapping pathways provide a coherent explanation for the strong associations between ALT and MetS observed across studies.

A particularly important finding in our study is the consistently stronger association of GGT with MetS compared with ALT, AST, and ALP. Although GGT showed comparatively larger effect‐size estimates than the other enzymes, this pattern should be interpreted as a potentially stronger association rather than diagnostic superiority, as no formal diagnostic performance metrics (AUC, PPV, and NPV) were evaluated. This pattern aligns with reports from China, Korea, and Iran [22]. While ALT, AST, and ALP typically rise in response to hepatocellular or cholestatic injury—such as fatty liver, steatohepatitis, or cholelithiasis [33], mechanistically, GGT plays a central role in glutathione turnover and extracellular antioxidant defense; elevated levels therefore reflect systemic oxidative stress and chronic low‐grade inflammation, two key mechanisms driving insulin resistance and metabolic dysfunction [34, 35] [6, 7]. This broader biological footprint likely explains GGT′s superior performance as a metabolic biomarker [22, 34, 35]. This dual hepatic and systemic relevance likely explains why GGT consistently outperforms ALT, AST, and ALP across diverse populations.

Sex‐related differences have also been reported. Pei et al. noted that GGT is more strongly associated with MetS in men, whereas ALT is more predictive in women [12]. Similarly, Iranian data suggest greater predictive value of high‐normal ALT and AST in women [26]. Although we initially aimed to explore potential sex‐specific patterns, our dataset did not include sex‐stratified enzyme distributions, preventing us from computing mean levels or prevalence of abnormality separately for men and women. Consequently, we were unable to evaluate enzyme–sex interactions in adjusted models. This limitation underscores the need for future cohort studies to incorporate sex‐stratified biochemical profiling to improve risk stratification. Although GGT showed comparatively larger effect‐size estimates than the other enzymes, this pattern should be interpreted as a potentially stronger association rather than diagnostic superiority, as no formal diagnostic performance metrics (AUC, PPV, and NPV) were evaluated.

Another notable insight, consistent with Aliabadi et al. [15], is the relatively weak association between AST and MetS, particularly within the normal range. Multiple investigations have shown that GGT and ALT are more strongly associated with MetS than AST [14, 22]. AST is predominantly a mitochondrial enzyme that typically increases in advanced hepatocellular injury, whereas ALT is more sensitive to early‐stage hepatic fat accumulation [36, 37].

Taken together, our findings indicate that liver enzymes particularly GGT and ALT may serve as practical, low‐cost biomarkers for identifying individuals at elevated metabolic risk. However, we did not assess diagnostic metrics such as AUC, PPV, or NPV, which are essential for determining clinical applicability. Future longitudinal studies should incorporate these measures and evaluate whether combining liver enzymes with other metabolic indicators improves predictive accuracy. This mechanistic distinction likely accounts for AST′s limited discriminatory capacity as an early metabolic marker.

## 5. Limitations and Strengths

Key strengths of this study include the large, well‐characterized cohort, standardized biochemical measurements, and adjustment for multiple confounding variables. Nonetheless, several limitations should be acknowledged. First, the cross‐sectional design precludes establishing causality or the temporal direction of associations. Second, we lacked imaging or histopathological confirmation of NAFLD. The third limitation is the absence of sex‐stratified liver enzyme data, which restricted our ability to examine descriptive or inferential sex‐specific patterns. Given the known physiological differences between men and women, future studies should collect and analyze enzyme distributions by sex. Fourth, we could not account for longitudinal fluctuations in enzyme levels. Although participants reporting significant alcohol use were excluded to minimize confounding—an important consideration since alcohol elevates GGT independently of MetS—underreporting is possible due to cultural restrictions, which may have led to residual confounding. Another limitation is the absence of formal diagnostic tests (AUC, PPV, and NPV), which precludes determining whether any enzyme genuinely outperforms others for clinical screening. Finally, while the sample is representative of the local adult population, generalizability to younger individuals or other ethnic and geographic groups is limited.

Despite these constraints, the study′s rigorous sampling procedures conducted under the PERSIAN Cohort framework and oversight of the Iranian Ministry of Health strengthen the reliability of our findings. In light of the anticipated global increase in metabolic abnormalities, which may place millions at a heightened risk of cardiovascular disease and Type 2 diabetes [38], incorporating simple biomarkers such as liver enzymes into population‐level screening programs could help reduce future healthcare costs and improve quality of life.

## 6. Conclusion

In conclusion, both elevated and high‐normal liver enzyme levels were associated with increased odds of MetS. GGT demonstrated higher ORs than the other enzymes, indicating a potentially stronger association with metabolic risk; however, this pattern should not be interpreted as a diagnostic superiority, as formal measures of diagnostic performance were not assessed. Liver enzymes—particularly GGT and ALT—may serve as accessible indicators for identifying individuals at an elevated metabolic risk, but their clinical application requires further validation. Future multicenter longitudinal studies should establish sex‐specific and population‐specific thresholds and evaluate external validity, diagnostic accuracy, and predictive values before incorporating these biomarkers into routine screening or risk‐stratification strategies.

**Nomenclature**
PERSIAN: Prospective Epidemiological Research Studies in IrAN
BKNCD: Bandare‐Kong Non‐Communicable Diseases
MetS: metabolic syndrome
NCEP: National Cholesterol Education Program
BMI: body mass index
CVD: cardiovascular disease
T2DM: Type 2 diabetes mellitus
BP: blood pressure
DBP: diastolic blood pressure
SBP: systolic blood pressure
FPG: fasting plasma glucose
TG: triglyceride
HDL‐C: high‐density lipoprotein cholesterol
AST: aspartate transaminase
ALT: alanine transaminase
ALK: alkaline phosphatase
GGT: gamma‐glutamyl transferase

## Author Contributions

H.S. designed and wrote the manuscript. M.K. supervised the study. E.S. performed the statistical analysis and interpreted the analyzed data. A.G. and S.M.H. wrote and revised the manuscript.

## Funding

No funding was received for this manuscript.

## Disclosure

All authors read and approved the final manuscript.

## Ethics Statement

The study obtained informed consent from all subjects. For the vulnerable population, informed consent was obtained from parents or legal guardians. The study protocol underwent thorough evaluation and received approval from the Ethics Committee of Hormozgan University of Medical Sciences (Ethics Code: IR.HUMS.REC.1401.310), aligning with the ethical principles outlined in the Declaration of Helsinki.

## Conflicts of Interest

The authors declare no conflicts of interest.

## Supporting information


**Supporting Information** Additional supporting information can be found online in the Supporting Information section. Sex‐specific normal reference ranges for liver enzymes based on the manufacturer′s standard kit described in Table S1.

## Data Availability

The datasets used and/or analyzed during the current study are available from the corresponding authors on reasonable request.
