# Supplementary material for: Association Between Liver Enzymes and Metabolic Syndrome: A Population‐Based Cross‐Sectional Study of the Bandar Kong Cohort Study
Source: Int J Hepatol. 2026 Apr 8;2026:8920064. doi: 10.1155/ijh/8920064 (PMC13059668; doi:10.1155/ijh/8920064)
Supplement: Supplementary file 1 — Supporting Information Additional supporting information can be found online in the Supporting Information section. Sex‐specific normal reference ranges for liver enzymes based on the manufacturer′s standard kit described in Table S1. [file IJH-2026-8920064-s001.docx]

The normal range for liver enzymes according to the standard kit reference

| **Liver enzymes** | **GGT** | **ALP** | **ALT** | | **AST** |  |
| --- | --- | --- | --- | --- | --- | --- |
| **Normal ranges** | Male <49 IU/L  Female<32 IU/L | Male: 80-306 IU/L  Female:64-306IU/L | Male: up to 41 IU/L  Female: up to 31 IU/L | Male: up to 37 IU/L  Female: up to 31 IU/L | | |

**Abbreviation:** **GGT**; gamma glutamyl transferase; **ALP**: alkaline phosphatase, **ALT**; alanine aminotransferase, **AST**; aspartate aminotransferase.
